# The relation between behavioral problems and social competence: A correlational Meta-analysis

**DOI:** 10.1186/s12888-019-2343-9

**Published:** 2019-11-09

**Authors:** Silje Hukkelberg, Serap Keles, Terje Ogden, Karianne Hammerstrøm

**Affiliations:** 1 The Norwegian Center for Child Behavioral Development (NUBU), Norwegian Research Centre (NORCE), Oslo, Norway; 2 Center for Child and Adolescent Mental Health, Oslo, Eastern and Southern Norway

**Keywords:** Meta-analysis, Children, Conduct problems, Social competence, Social skills

## Abstract

**Background:**

Previous studies have shown that children who display behavioral problems also tend to display low social competence. The relation does however vary according to type of behavior being measured, as well as demographic characteristics of the respondent. The present meta-analysis examined the correlation between different types of behavioral problems and social competence among children aged 3–13, and investigated possible moderators in this relation.

**Methods:**

A systematic literature search was conducted for English language studies from January 2008 to January 2018 that reported correlations between three types of behavioral problems (i.e., externalizing behaviors, conduct problems, or aggression) and two types of social competence (i.e., social competence or social skills). The studies included reports from parents and teachers, or both as a dyad. The review included data from 54 independent studies and a total of 46,828 participants. Effect sizes were estimated using a random effects approach and moderator analyses between subsets of categorical variables were tested by the significant Q test.

**Results:**

Results showed an overall correlation between behavioral problems and social competence of medium effect size (*r* = −.42, *p* < .01). Moderation analyses indicated no significant differences for different types of behavioral problems or social competence. However, a significant difference was found with regard to type of respondent; the correlation was significantly higher when both measures were reported by the same respondent (teacher or parent) compared to when measures were reported by parent-teacher as a dyad.

**Conclusions:**

Findings summarized and quantified a robust negative correlation between behavioral problems and social competence. The results indicate that intervention programs targeting problem behaviors in children would benefit from reducing behavioral problems and in concert, increase social competence to help children with emerging or present problem behaviors.

## Background

Children with behavioral problems seem to have an increased risk for entering a negative developmental pathway in which they experience e.g., high levels of academic failure, depression/anxiety, eating disorder, as well as interpersonal and health related problems ([19, 31]; Jaffee, Strait, & Odgers, 2012; [51, 54, 55, 62, 70, 75]). Especially, the relation between social competence and behavioral problems has attracted attention as it has turned out that the promotion of social competence in children and youth may actually be a viable alternative or supplement to efforts at reducing these problems.

Over the past decade, a number of studies, linking behavioral indicators and interpersonal challenges, have reported an inverse relation between behavioral problems and social competence in children; that is, high levels of problems seem to associate with low levels of social competence, or vice versa ([4, 17]; Montroy, Bowles, Skibbe, & Foster, 2014). Findings suggest that poor social competence, which often include difficulties with social information processing, problems with adapting to a situation, and rejection by friends, may contribute to the development and maintenance of behavior problems (Coie & Dodge; Loeber & Ferrington, 2001). However, as it stands, the magnitude of this relation is still unclear, as the correlation varies substantially across studies ([25, 30]; Ren, Zhang, Zhou, & Ng, 2017). Apparently, not all children who show poor social competence exhibit behavioral problems, and not all children who display these problems are socially inept. It is of great interest to understand the strength and nature of this correlation to reveal how these two concepts are connected, in order to develop effective early intervention programs for children with behavioral problems and accompanying impairment in social functioning. Thus, a meta-analysis is warranted, to systematize the findings of the extant studies in literature that have investigated the relation between behavioral problems and social competence, and to examine the overall strength of this correlation as well as the conflicting results across studies. Hence, the current meta-analysis by summarizing extant studies on the relation aims at filling this gap. In addition, we examined possible moderators related to the conceptualization of constructs, assessment, and respondents that may explain the variation found in the strength of this correlation (i.e., heterogeneity) among studies.

### Social competence and social skills

While some children easily navigate social encounters, other children lack the ability or motivation to interact with peers and adults in a positive way. Social impairment can be displayed in different ways. Disruptive children seem to struggle with emotion regulation, internalization of rules, are slow to develop empathy and conscience, and often lack adaptive problem solving skills [74]. For example, children may interact with others in an aggressive and disruptive way, and over time, social impairment increases the likelihood of rejection and disliking by peers [22]. Thus, social impairment seems to have short and long-term ripple effects encompassing poor social- and behavioral adjustment. Social competence deficits thus often limit the possibilities for future interactions and limit further skill development [35].

Typically, the ability to negotiate age-appropriate social encounters is measured by either social competence or social skills. These constructs tend to be overlapping and somewhat ambiguously defined, as both often cover indices that relate to language, intelligence, attitude, and interaction with the environment [5]. In addition, both constructs are characteristically dynamic as children’s social competence and social skills develop over time. That is, as children age, they encounter new developmental tasks with increasing complexity, and their social interplay gradually moves from social interaction with parents and siblings at home, to spending more time with peers in kindergarten and at school, where peer relations are considered most important in meeting unique and complex social skills [11]. However, to date, there is no agreed upon definition of social competence and social skills, which has resulted in an ongoing controversy about the nature, conceptualization and measurement of these constructs (e.g., Nangle, Grover, Holleb, Cassano, & Fales, 2000; Rose-Krasnor, 1996).

Social competence is a broadly adaptive characteristic and can be defined as “the ability to take another’s perspective concerning a situation and to learn from past experiences and apply that learning to the ever-changing social landscape” (page 1, [67]). In essence, the definition captures a child’s awareness of how one’s behavior affects his/her surroundings and sensitivity to the needs of others. As such, manifestations of social competence are multifaceted and may include friendship, popularity with peers, positive self-concept, social assertion, and so on ([23, 71]. Although the distinction between social competence and social skills is sometimes overlooked, social skills generally refer to specific abilities or behaviors that are needed to perform a task [52]. Social skills describe the ability to accurately select relevant and useful information from a social context, and use this information to explore opportunities for goal attainment and maintain good relationships with others. Social skills are both cognitive and interpersonal, verbal and non-verbal conditions for appropriate social behavior and positive social interactions [5].

Altogether, social competence could be thought of as a more general and evaluative term, whereas social skills are more situation specific behaviors [32] or responses [60]. However, although individual skills contribute to overall social competence, no single behavior is sufficient for social competence (Hupp, LeBlanc, Jewell, & Warnes, 2009), and social competence in one situation not necessarily transfers to other situations [52].

### Behavioral problems: externalizing behaviors, conduct problems, and aggression

Pervasive and persistent behavioral problems in children and youth are considered as a risk factor for successful functioning in different arenas; at home, at school, and among peers [29, 47]. Furthermore, these problems are also associated with detrimental future consequences, including antisocial behavior, social exclusion, and severe psychopathology ([16, 57]. In a diagnostic approach, conduct disorder and oppositional defiant disorder are the most prevalent diagnoses (DSM-V; ICD-10). However, since there are no corresponding diagnoses for social competence deficits, comparisons between conduct disorders and social competence becomes impossible. We have therefore excluded studies that exclusively use a diagnostic approach.

Research suggests that behavioral problems as early as age three may predict affiliation to a population segment representing high future costs in adulthood [16]. Studies on social competence - behavioral problems relation may also vary according to the range of the problem construct being measured. In this study, we limit our focus on three different types of behavioral problems: externalizing behaviors, conduct problems, and aggression. The terms externalizing behaviors (i.e., negative behaviors that are directed toward the external environment [12];) and antisocial problems are often used synonymously, yet the externalizing behaviors is often used to describe less severe disruptive and destructive child behavior [26, 68]. Conduct problems, on the other hand, are generically defined as high rates of aggression, noncompliance, oppositional behaviors [76], and typically assessed with the Achenbach’s Child Behavior Checklist [1, 46]. Finally, although there are no definite agreement about the definition of aggression [37], it is often considered a subset of broader concepts (e.g., externalizing behaviors and conduct problems [26];). Typically, aggression covers physical and verbal behavior directed towards individuals with an intent to harm (e.g., pushing, kicking, and threatening; APA, 1994). Most studies of aggression have focused on physical aggression, although other types have also been described, like for example indirect [15] and relational aggression [21] that reflect different forms and functions of aggression ([14, 50]. Studies show that childhood aggression is a strong predictor of adult crime and violence [26, 56]. In the current article, the concepts of externalizing behaviors, conduct problems and aggression represent independent measures that cover from a broad to a narrow range of behavioral problems that may possibly correlate differently with social competence.

### Purpose of this study

To summarize, social competence in childhood has become an area of interest for both researchers and clinicians, because of its negative correlation with behavioral problems (or vice versa), and the important role it seems to play in shaping future adjustment abilities in youth and adulthood. From a clinical perspective, effective treatment and prevention of behavioral problems in children should be comprehensive and address both risk and protective factors. That is, on the one hand, risk factors for the development of these problems should be reduced, and on the other, efforts at promoting social competence should simultaneously be encouraged.

The relation between social competence and different manifestations of behavioral problems is, however, not obvious, given the variation in the strength of this relation among numerous studies (e.g., Burt, Obradović, Long, & Masten, 2008 [30, 64];). Due to lack of research examining moderating variables that may explain variations among studies, a meta-analysis is warranted. The main aim of this study was to review and systematically examine studies testing the correlation between behavioral problems and social competence among children, as well as to investigate the role of various moderators of the reported correlations. With this aim, we made a distinction between social competence and social skills, and between types of behavioral problems as externalizing behaviors, conduct problems, and aggression. Furthermore, we investigated if the variations in correlations would be moderated by characteristics of study design and participants. We limited our analysis to samples of preschool and school age children, when problem behavior is more likely to be malleable, and can be successfully changed through interventions programs. Therefore, the relation between behavioral problems and social competence is especially interesting for this age span to enhance our knowledge and develop interventions.

## Method

**Fig. 1:**
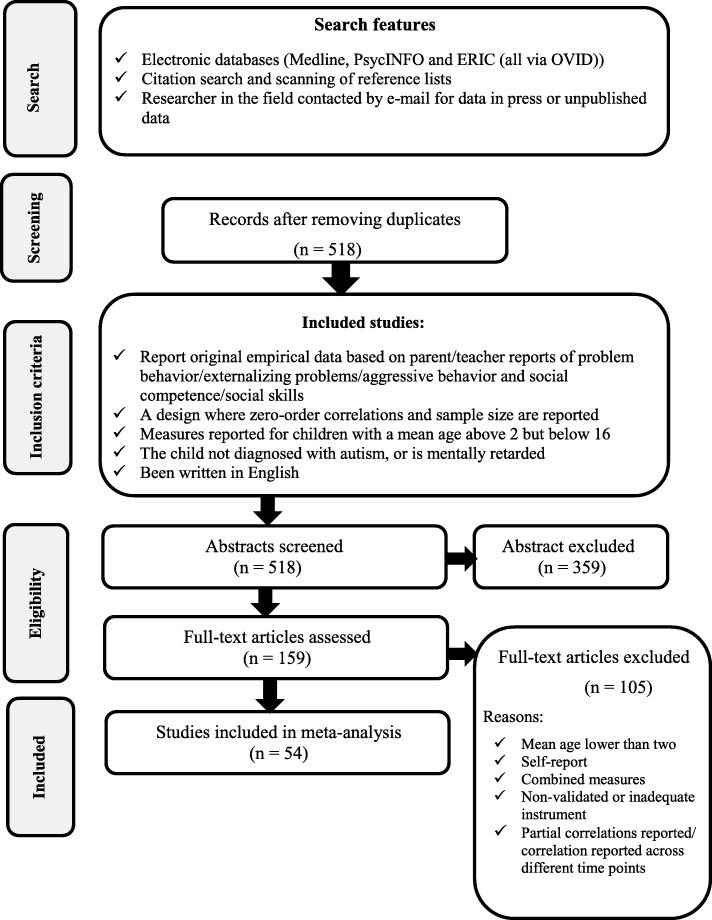
Flow diagram for studies included in the meta-analysis

Meta-analysis was performed in accordance with the Preferred Reporting items for Systematic Reviews and Meta-Analyses (PRISMA) statement [58]. The flow diagram of the various steps in the analysis is depicted in Fig. 1.

### Literature search

We conducted a search in Medline, PsycINFO and ERIC (all via OVID) in June 2018. The search strategy included the terms social competence, social skills, and interpersonal. These were combined by the boolean operator ‘AND’ with terms describing antisocial, problem behavior, aggression or conduct disorder, as well as terms describing children or adolescents (see search strategies in Additional file 1).

The search was limited to papers published in 2008 or later, and restricted to papers in English, in Scandinavian languages or in Turkish. We conducted a manual search of the references in the studies we identified. Finally, in order to access unpublished data, we contacted researchers in the field by e-mail for data “in press” or for unpublished data. Applying the inclusion criteria below, we examined titles, abstracts or full text for relevance.

### Inclusion criteria and coding

For inclusion in the present meta-analysis, studies had to meet the following criteria: (1) reporting correlation and sample size for the relation between social competence and conduct problems, (2) for children and adolescents 3 > age < 13, (3) measured social competence or social skills, (4) measured antisocial behaviors, externalizing behaviors, conduct problems, or aggression, (5) included reports by parent(s) and/or teacher(s), (6) conducted between January 2008, and January 2018, (7) published in English, Scandinavian languages or Turkish. Accordingly, studies were excluded from the analysis if correlations (1) were based on several instruments combined, (2) included pooled responses from different respondents (e.g., average correlation of self and parent reports) (3) were from studies focused on children with co-occurring problems such as ADHD, learning disabilities, and anxiety.

The search resulted in 518 potentially studies, whereof 54 were identified as independent and relevant studies, in accordance with the inclusion criteria. See Table 1 for a list of the included studies with selected descriptives. Each of the selected studies was coded at least twice with the following variables: (1) publication year, (2) correlation and sample size (N), (3) type of social competence measure: whether the study reported social competence or social skills, (4) type of conduct problems: whether the study reported on externalizing behaviors, conduct problems, or aggression, (5) type of instrument: whether the same instrument used to measure both constructs or not, (6) type of respondent: parent vs. teacher or parent and teacher as a dyad, (7) mean age: the average age of participants, in addition as a categorical moderator, i.e., age < 6 and age ≥ 6 (school-age), (8) gender as percentage of females, (9) country of study origin: USA vs. Europe vs Asia and (10) socio-economic status of the sample. When fewer than five studies reported data on a moderator, the variable was excluded from further analysis. Consequently, we were not able to perform country of study analysis on studies from Australia (*N* = 2), Canada (*N = 1*), or Africa (*N* = 1). We were able to retrieve data from three unpublished studies ([44]a, b [73];), and thus analysis was not performed on published vs non-published data. In order to prevent violation of independence of observations (i.e., including data from the same sample several times), the correlation of the first assessment was included in the analysis, when correlations for several time-points were reported. However, the second wave of observation was used in cases when the first assessment was based on children of age two or below, in line with the inclusion criteria. Further, in order to represent all categories, we made some choices, i.e., parent-teacher reports were prioritized before other reports, aggression reports were used over other reports, and conduct problems reports were used before externalizing behaviors reports.

**Table 1:** Selected descriptives and effect sizes for included studies

Study	Sample	Mean age	% girls	Measure	r	*N*
Ansari, 2018	USA	4.0	51.0	EXT/ SS	−.65	15,070
Arnold et al., 2012	USA	4.7	48.2	AGG/ SS	−.38	467
Baker et al., 2015	USA	4.6	50.0	CP/ SS	−.46	760
Barnett et al., 2010	USA	3.5	52.0	EXT/ SC	−.32	127
Bjørknes & Manger, 2013	Norway	5.9	37.0	EXT/ SS	−.59	96
Blandon et al., 2010	USA	5.8	54.0	CP/ SS	−.67	253
Brock & Curby, 2014	USA	5.1	48.0	CP/ SC	−.70	2938
Broekhuizen et al., 2015	Netherlands	3.0	46.6	EXT/ SC	−.45	545
Buck, 2014	USA	6.5	52.0	EXT/ SS	−.20	1022
[17]	USA	7.4	48.1	EXT/ SC	−.23	258
Chen et al., 2010	China	8.3	48.7	AGG/ SC	−.50	1140
Chen et al., 2011	China	7.7	55.5	EXT/ SC	−.15	425
Denham et al., 2013	USA	4.5	50.0	AGG/ SC	−.34	298
Dollar & Stifter, 2012	USA	4.5	47.8	AGG/ SC	−.35	90
Engle et al., 2011	USA	3.0	48.9	EXT/ SS	−.55	567
Erturk, 2017	Turkey	3.5	52.8	CP/ SS	−.55	53
Gresham et al., 2011	USA	9.2	54.1	EXT/ SS	−.47	146
Hoglund et al., 2015	USA	8.2	50.1	AGG/ SC	−.74	941
Hosokowa & Katsura, 2017	Japan	6.1	48.5	EXT/ SS	−.44	1604
Huang et al., 2017	Uganda	6.5	50.7	CP/ SC	−.24	303
Hubbard et al., 2013	USA	10.5	52.0	AGG/ SC	−.18	594
Hukkelberg et al., 2018a	Norway	7.3	31.9	CP/SC	−.55	216
Hukkelberg et al., 2018b	Norway	8.4	36.5	CP/SC	−.61	137
Jia et al., 2012	USA	4.0	48.2	EXT/ SC	−.31	112
Kim et al., 2010	Korea	6.1	63.2	CP/ SC	−.30	76
Korucu et al., 2017	Turkey	4.5	0.5	AGG/ SC	−.40	212
Li et al., 2015	China	4.6	44.3	EXT/ SS	−.29	543
Main et al., 2017	USA	9.2	48.1	EXT/ SC	−.36	238
Marti et al., 2016	USA	3.8	51.0	EXT/ SC	−.12	106
Mihic et al., 2016	Croatia	4.5	45.0	AGG/ SC	−.40	182
Mirabile, 2014	USA	4.5	53.0	EXT/ SC	−.61	81
[59]	USA	4.1	33.9	CP/ SS	−.72	118
Nix et al., 2016	USA	4.0	54.0	AGG/ SC	−.78	356
Orta et al., 2013	Turkey	4.6	42.4	EXT/ SC	−.47	118
Pasiak & Menna, 2015	Canada	4.6	23.7	AGG/ SS	−.67	59
Perry-Parrish et al., 2012	USA	6.5	47.0	EXT/ SC	−.16	523
Pluess & Belsky, 2009	UK	4.5		CP/ SC	−.62	968
Razza & Raymond, 2013	USA	4.5	49.2	EXT/ SS	−.55	1007
Razza et al., 2015	USA	9.0	52.2	EXT/ SS	−.53	669
Ren, 2014	Australia	4.3	46.0	AGG/ SS	−.40	100
Rich, 2008	USA	4.0	48.1	AGG/ SS	−.49	77
Roberts et al., 2016	USA	4.0	49.1	CP/ SS	−.13	2203
Sette et al., 2015	Italy	4.6	51.1	AGG/ SC	−.35	493
Sette et al., 2017	Italy	4.7	51.8	EXT/ SC	−.31	112
[69]	Iceland	8.0	27.0	CP/ SS	−.15	102
Skalicka et al., 2015	Norway	4.0	50.0	EXT/ SS	−.19	981
[73]	Norway	11.5	49.4	EXT/SS	−.52	8013
Torres et al., 2014	Portugal	4.5	52.8	AGG/ SC	−.41	295
Valiente et al., 2011	USA	6.1	44.9	EXT/ SC	−.23	214
Veiga et al., 2016	Portugal	5.8	53.8	EXT/ SC	−.23	78
Wildenger & McIntyre, 2012	USA	5.4	59.3	CP/ SS	−.11	86
Wilson et al., 2012	Australia	4.2	48.0	CP/ SC	−.03	128
Zhang, 2012	China	3.5	53.9	EXT/ SC	−.48	103
Zhou et al., 2015	China	7.7	55.5	EXT/ SC	−.15	425

*Notes. EXT* Externalizing behaviors, CP = Conduct problems, AGG = Aggression, SC = Social competence, SS = Social skills

To ensure reliable coding of the moderators, the first two authors (SH and SK) together generated a coding system for the moderators as well as other study characteristics (e.g., type of the outcome measure) and coded each study separately. Interrater agreement was calculated by dividing the total number of congruent observations to the total number of observations and multiplied times 100. Interrater agreement rate was 98.5%. Finally, the two first authors held consensus meetings, to resolve inconsistencies by consulting the article or by discussion, to reach 100% agreement.

### Statistical analyses

#### Effect size calculation

The analyses were conducted using the Comprehensive Meta-Analysis program, Version 3 (CMA [7];). Descriptive analyses were performed in Microsoft Excel. The Pearson correlation coefficient (*r*) was used as the effect size for this meta-analysis, together with sample size (N), for each study. Overall effects (*r*) were transformed to Fisher’s *z* [27] for analyses, and then converted back to the correlation coefficients to ease comparability (i.e., a weighted effect size). Further, a 95% confidence interval (CI) was calculated for each effect size, to examine whether each effect size was significantly larger than zero. We used the benchmark values offered by Cohen [18], i.e., r of .10 was considered a small effect size, whereas r of .30 of a medium effect, and r of .50 or above as a large effect size. Separate effect sizes were calculated for moderator analysis, and differences between correlations were statistically tested. Random-effects model was employed, anticipating that the true effect size varies among studies (Borenstein, Hedges, Higgins, & Rothstein, 2009), and allows the results to be generalized beyond the selected studies [8]. Forest plots were used to inspect the distribution of effect sizes, and identify possible outliers. Sensitivity analyses were employed to detect the impact of outliers.

#### Effect size heterogeneity

Variation, or heterogeneity, in effect sizes between studies, were considered by the means of *Q* statistic [39] that reveal if there is a significant variability among each set of effect sizes, larger than what could be expected from sampling error only [49]. In addition, *I*^*2*^ values were reported, which show the total percentage of variability in a set of effect sizes arising from between-study differences [40]. The *I*^*2*^ value ranges from 0 to 100%, in which lower values are thought to reflect spurious observed variance, whereas larger values are thought to reflect more serious reasons for the observed variance [8], and consequently reasons to perform moderator analyses. Established benchmark values of *I*^*2*^ are 25, 50, and 75%, representing “low,” “moderate,” and “high” values, respectively [41].

#### Moderator analyses

Moderator analyses investigating the effects of child’s age and gender, SES background, origin of study, publication year, and type of measurement, instrument, and respondent to explain the heterogeneity across studies. We used the corrected correlations (r) with random effects models. A minimum of 5 studies was required to consider a moderator as usable. For the continuous moderator variables (e.g., percentage of girls), we used meta-regression based on the method of moments for random-effects models to predict variations in effect size across studies from the moderator variables.

#### Publication Bias

In order to detect retrieval bias, funnel plots for random-effects models were examined. Here, the sample size was plotted on the y- axis and effect size on the x- axis and in the absence of retrieval bias, the plot was expected to form an inverted funnel. In the presence of bias, the funnel presented shows an asymmetric distribution. If results indicated publication bias, the “trim-and-fill” procedure [24] was followed, to get an estimate of the impact of publication bias on the meta-analysis results.

## Results

### Study characteristics

Fifty-four non-overlapping samples were identified, and included in the present meta-analysis. The study selection process is presented in Fig. 1.

The characteristics of all studies were displayed in Table 1.The total sample size was 46,822 participants (range: 53–15,070), with a mean number of 867 participants per study. The majority of samples came from USA (*N =* 27, 50%), followed by Europe (*N =* 16, 29%), Asia (*N =* 7, 13%), Australia (*N =* 2, 4%), Canada (*N = 1*, 2%) and Africa (*N = 1*, 2%). Mean age of children was 5.62 years (*SD* = 1.97), and the percentage of girls ranged from 23.7 to 63.2% (*M* = 47.37, *SD* = 9.62).

### Overall correlation between social competence and behavioral problems

**Fig. 2:**
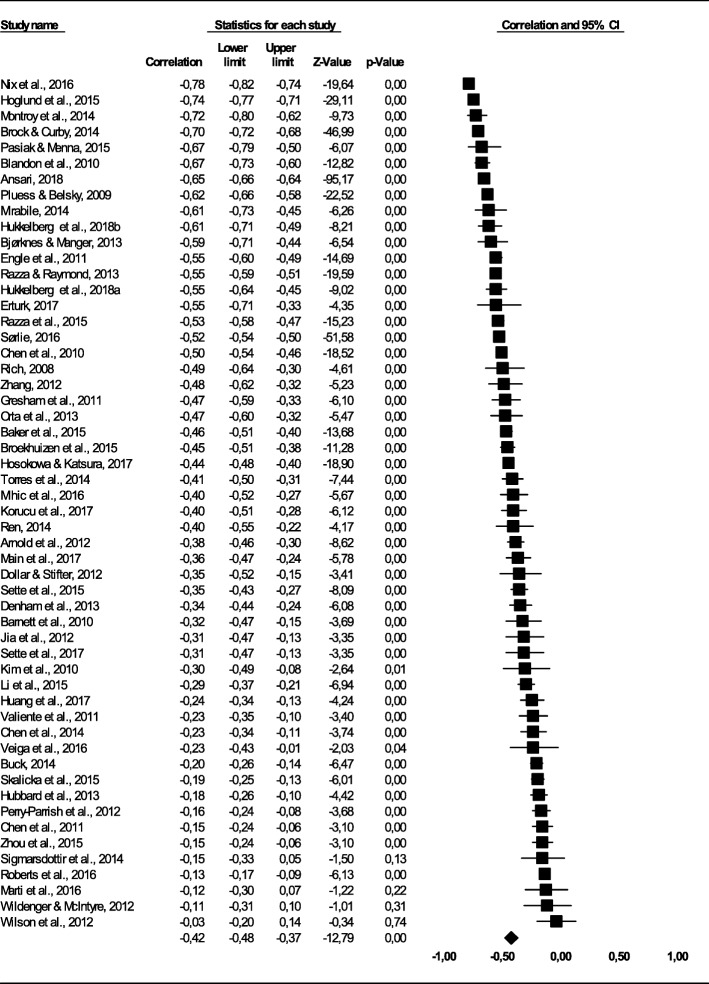
Forest plot showing effect size (sorted from low to high) with confidence interval, z-value, and *p*-value for the correlation between conduct problems and social competence for each study, and the overall correlation (black diamond) across studies

Figure 2 shows the forest plot with effect sizes and confidence intervals for each sample (k = 54), in addition to the pooled result, calculated with the random effects weights. The average corrected correlation between social competence and behavioral problems was negative and significant (*r* = − .42 [95% CI = − .48,-. 37], *Z* = − 12.79, *p* < .01). That is, overall higher levels of social competence were associated with lower levels of behavioral problems. However, the variation in correlations across samples was significant and high (*Q* = 2529.01, *p* < .001) and the I^2^ statistics showed that 97.90% of the observed variability was beyond what could be expected by chance. This not only justifies using the random effects model, but more importantly, it indicates that it is likely to be one or more variables that moderate the relation between social competence and behavioral problems.

The funnel plot for correlations was close to symmetrical, but indicated that 5 studies were missing to the left of the mean. In a trim and fill analysis these five studies were added, which adjusted the overall correlation to *r* = −.45, [95% CI = − .50,-. 40]. The adjusted correlation was still significantly different from zero, suggesting that publication bias is not affecting the results considerably. Following [36], removing the samples with the highest (Nix et al., 2016; *r* = −.78) and the lowest (Wilson, Havighurst, & Harley, 2012; *r* = −.03) effect sizes did not reduce the Q statistic with more than 50% (*Q* = 2416.68), nor did removing the study with the largest sample size (Ansari, 2018; *N* = 15,070, Q = 1776.22). Consequently, all of the 54 study samples were included in the meta-analysis.

### Moderator analyses

Due to the heterogeneity of the overall effect sizes in the relation between social competence and behavioral problems, moderator analyses were conducted. Method-related moderators and demographic moderators were analyzed. Table 2 presents the examined categorical moderators and test statistics.

**Table 2:** Overall analyses and subgroup analyses

Overall effect	*k*	r	95% CI	I^2^	Q_B_	Q_W_
	54	.42	[−.48, −37]	97.90	2529.01**	
Moderator analyses						
Methods-related moderators						
Type social						
Social competence	32	−.40**	[−.48,-.32]	97.09		1066.56**
Social skills	22	−.46**	[−.54,-.36]	98.55		1446.75**
					0.88^ns^	
Type conduct problems						
Aggression	14	−.48**	[−.58,-.36]	96.52		373.84**
Conduct problems	14	−.45**	[−.56,-.33]	98.47		847.79**
Externalizing	26	−.38**	[−.47,-.29]	98.05		1280.29**
					1.95^ns^	
Instrument						
Same	19	−.45**	[−.54,-.36]	96.77		557.44**
Different	35	−.41**	[−.47,-.34]	97.25		1237.55**
					0.69 ^ns^	
Respondent						
Parent-parent	12	−.47**	[−.55,-.38]	88.62		96.65**
Teacher-teacher	28	−.51**	[−.56,-.46]	97.13		942.02**
Parent-teacher	14	−.18**	[−.27,-.09]	42.88		22.76**
					42.72**	
Demographics-related						
Age						
Age < 6	35	−.45**	[−.52,-.38]	97.93		1644.95**
Age ≥ 6	19	−.37**	[−.47,-.27]	97.18		637.81**
					1.68 ^ns^	
Study continent						
Asia	7	−.34**	[−.50,-.15]	93.58		93.47**
Europe	16	−.44**	[−.54,-.32]	92.62		220.65**
USA	27	−.45**	[−.53,-.36]	98.59		1842.76**
					1.38^ns^	
SES						
Low	16	−.45**	[−.55,-.34]	98.76		1212.81
Middle/High	38	−.41**	[−.48,-.34]	97.13		1289.60
					.29^ns^	

_*p < .05, **p < .01,_ *Q*_*B* = homogeneity statistic *Q* between groups;_ *Q*_*W* = homogeneity statistic *Q* within groups_

#### Method-related moderators

##### Differences by construct bandwidth and instrument type

Thirty-two studies (*N =* 27,902) reported a correlation based on a social competence measure, whereas 22 studies (*N = 18*,926*)* reported a correlation based on a social skills measure. The overall weighted effect size for the relation between social competence and behavioral problems was *r* = −.40 (95% CI [− .48,-.32], *p* < .01), and the average correlation for studies based on social skills was *r* = −.46 (95% CI [− 0.54, − 0.36], *p* < .01). There was no significant difference in the overall correlation across social competence measurements *Q*(1)_between_ = 0.88, *p* = .35.

For behavioral problems, we coded three different constructs with decreasing bandwidth: externalizing problems, conduct problems, and aggression. One study (Buck, 2014) reported antisocial behavior, and this study was categorized as externalizing behavior as these constructs are used synonymously [42]. Twenty-six studies (*N* = 33,183) examined the correlation between social competence and externalizing problems. The overall mean correlation among these studies was *r* = −.38 (95% CI [− .47,-. 29], *p* < .01). Fourteen studies comprising 8341 children examined the correlation between social competence and conduct problems. The overall mean correlation between conduct problems and social competence was *r* = −.45 (95% CI = [−.56,-.33], p < .01). Finally, fourteen studies (*N =* 5304) investigated the relation between social competence and aggression. The average effect size between social competence and aggression was *r* = −.48 (95% CI [− .58,-. 36], *p* < .001). Overall, the between-level Q appeared non-significant *Q(2)*_between_ = 1.95, *p* = .38, suggesting that the correlations between social competence and behavioral problems was not moderated by the type of problems being measured. Moreover, we tested whether relying on the same instrument (e.g., SSRS or HSCBS) when assessing both social competence and behavioral problems was advantageous compared to when using different instruments in the assessment of the two constructs. Results showed that instrument type does not moderate the overall correlation (*Q(1)*_*between*_ = 0.69*, p* = .41*).*

##### Type of respondent

To examine whether type of respondents moderated the overall correlation, three different dyads were analyzed: parent-parent report (k = 12, *N* = 3842), teacher-teacher report (k = 28, *N* = 36,163), and parent-teacher report (k = 14, *N* = 6823). The average correlations were *r* = −.47 (95% CI [− .55,-. 38], *p* < .01) for parent reports, *r* = −.51 (95% CI [− .56,-. 46], *p* < .01) for teacher reports, and *r* = −.18 (95% CI [− .27,-. 09], *p* < .01) for parent-teacher as a dyad. The results revealed that the correlation between social competence and behavioral problems was significantly different across respondent groups (*Q(2)*_*between =*_ 42.72, p < .01). That is, it was substantially higher when both social competence and behavioral problems were reported by the same respondent, compared to when these problems was reported by parent and social competence was reported by teacher. Additional analyses showed that parent and teacher reports were not significantly different from each other (*Q(1)*_*between =*_ 0.77, *p = .38*).

##### Year of publication

In order to investigate whether the relation between social competence and behavioral problems was influenced by the year of study publication, we ran a meta-regression analysis. Year of publication was not a significant predictor of the overall effect size (intercept = − 15.84, slope = −.008, *SE* = 0.015, *Z* = −.56, *p* = .58, k = 54, R^2^ = .00).

#### Demographic moderators

##### Age and gender

In order to investigate if child age moderates the relation between behavioral problems and social competence, we considered age as both a categorical and continuous moderator. When considering age as a categorical variable, we made one category representing children below 6 years (k = 35, *N* = 29,782), and another category for those equal or above 6 years (k = 19, *N* = 17,046), reflecting preschool and school age, respectively. Results showed that the correlation was higher in the youngest group (*r* = −.45 (95% CI [− .52,-.38], *p* < .01) versus the eldest group *r* = −.37 (95% CI [− .47,-.27], *p* < .01). Between-level Q analysis revealed that age group did not moderate the overall correlation between social competence and behavioral problems (*Q(1)*_*between*_ = 1.68, *p* < .20). In addition, we investigated age as a continuous moderator using meta-regression. Age appeared as a non-significant predictor of the effect size (intercept = − 0.51, slope = .01, *SE* = 0.019, *Z* = 0.49, *p = 0.62,* k = 54, R^2^ = .00), which shows that the correlation between behavioral problems and social competence is stable with age covered in this study.

Percentage of females in each sample was coded, and gender was therefore examined as a continuous variable. One study (Pluess & Belsky, 2009) did not report percentage of girls, and was therefore excluded from this analysis. Results showed that percentage of females was not a significant predictor of the effect size (intercept = − 0.63, slope = .004, *SE* = 0.004, *Z* = 1.01, *p = 0.31*, k = 53). A R^2^ = .00 also confirms that gender per se did not have an impact on the overall effect size.

##### Continent and socioeconomic status

Since only one study was from Canada and Africa, in addition to two studies from Australia, we were not able to use these continents in a between-study comparison. Analyses were performed among USA, Europe and Asia (k = 50), and results showed the following effect sizes for the three continents (USA: *r* = −.45 (95% CI [−.53,-. 36], *p* < .01; Europe: *r* = −.44 (95% CI [− .54,-. 32], *p* < .01; Asia: *r* = −.34 (95% CI [−.50,-.15]). Continent did not moderate the overall correlation between social competence and behavioral problems (*Q(2)*_*between*_ = 1.38, *p* = .50). Additional analyses showed that neither the correlations between USA and Asia (*Q(1)*_*between =*_ 1.16, *p = .28*), USA and Europe (*Q(1)*_*between =*_ 0.032, *p = .86*), nor Europe and Asia (*Q(1)*_*between =*_ 2.08, *p = .15*) were significant.

Socioeconomic status (SES) was categorized as low versus middle/high, reflecting that the risk for developing conduct problems and poor social adjustment seem to increase with addition risk factors, like low SES [20, 66]. Results showed a small mean weighted effect size was found between studies, and the between-level Q did not moderate on the overall correlation between social competence and behavioral problems (*Q(1)*_*between*_ = .29, *p* = .59).

## Discussion

The purpose of this meta-analysis was to provide an estimate of the strength of the correlation between behavioral problems and social competence among children and adolescents in the literature. Overall, our results showed that the relation was negative and significant of medium effect size (*r* = −.42, *p <* .001). Consequently, children who display high levels of behavioral problems tend to show lower levels of social competence, or vice versa. This finding has both theoretical and practical implications. Theoretically, the moderate correlation suggests that these two constructs should be thought of as separate but related dimensions under the overarching concept of social functioning, a finding that has also been noted by others [13, 72]. Practically, the result indicates that intervention programs targeting behavioral problems should, in addition to these problems, also encourage social skills and social competence.

Nevertheless, it is worth noting that our results reflect a reciprocal negative relation between social competence and behavioral problems, and it is extremely difficult to disentangle the direction of the effect between these constructs. The extent to which lack of social competence causes higher levels of behavioral problems, or high levels of these problems impairs social competence represent a paradox of the hen and the egg. However, Bornstein and colleagues [9] found that children with lower social competence at age four years exhibited more externalizing and internalizing behaviors at age 10 years and more externalizing behaviors at age 14. However, we cannot dismiss that the correlation between the two constructs may be caused by a third, common factor. This may be a genetic factor, like personality traits (e.g., callous-unemotional traits; Frick, Ray, Thornton, & Kahn, 2014) that are innate or activated during early development, or an environmental factor such as bullying especially in the school context. Moderator analyses were, however, able to shed light on several interesting findings about the variation in the strength of social competence - behavioral problems relation, and helped us to better understand the heterogeneity of the results across studies reporting this relation.

### Differences by social competence and behavioral problems constructs

Our findings suggested that whether studies used the broadly defined social competence construct, or the more narrowly defined social skills, did not change the magnitude of the effect size. That is, assessment of social competence vs. social skills was not able to explain variations in the strength of the correlation among studies. However, this finding may reflect the present controversy pinpointing the often-poor discriminatory power in the assessment between these two constructs. That is, constructs are often measured with overlapping or similar items that capture such as communication and interpersonal skills. Although context and indices together combine to form the different constructs of social competence and social skills, these are, by definition, thought to give diverse outcomes. Social skills in one setting do not necessarily imply social skills in another setting, or general social competence [45, 52]. However, several theories of social competence depict a hierarchical relation between social competence and social skills. Gresham and Elliott [33] suggest that social skills and adaptive behavior represent two subdomains under the construct of social competence. Although social competence is considered as a more overarching term, it is also measured in a context (e.g., by teachers at school), and may as such be difficult to separate from social skills. The present results indicate that it does not matter whether one relies on social competence or social skills, at least when studying how these constructs relate to conduct problems. Nevertheless, this result does not dismiss the fact that researchers should be precise and conscious about what construct they are using. It may be that whether one relies on social competence or social skills make a difference in relation to other child behaviors.

The specificity or range of behavioral problems, also, did not moderate the correlation between social competence and these problems. That is, whether externalizing behavior, conduct problems, or aggression were measured, it did not have a significant influence on the magnitude of the effect size. One possible explanation may relate to the high correlation between the various constructs measuring these problems [10], especially in older children. Even though many young children, especially boys, may express normative aggression ([3, 19, 38]; Nærde, Ogden, Janson, & Zachrisson, 2014), a small number of children express persistent and high levels of aggressive behavior later on [63]. These children also show an increased risk for behavioral problems, as aggression takes a broader form. However, most studies in this analysis reported the measure of externalizing problems, while fewer reported on aggression and conduct problems. Thus, it may be that the results would be different, if we had more reports for the two latter constructs. Overall, present results indicated a robust moderate negative correlation between social competence and behavioral problems, independent of the bandwidth of constructs.

Furthermore, some measurement instruments assess both social competence and behavioral problems using the same format across constructs (e.g., HCSBS [53]; or SSIS [34];). In the present study, however, most studies reported using different instruments when assessing social competence and behavioral problems (*N* = 35). The results indicated that type of instrument did not moderate the overall negative correlation, thus the instrument format does not have an influence on the results.

### Respondents as moderators

The type of respondents appeared as a significant moderator of the social competence - behavioral problems correlation. Our results suggested that this correlation was lower when the two constructs were reported by different respondents, compared to same reporter for the both constructs. That is, the relation was low and non-significant when parents reported on behavioral problems and teacher reported on social competence, whereas high and significant when parents or teachers reported on both constructs (*r* = −.47 for parent reports, and r = −.51 for teacher reports, both significant at *p* < .001). The low correlation between parent-teacher as a dyad (*r* = −.18, *p* < .001) probably reflects the fact that parents and teachers evaluate behaviors in different contexts, at home and at school, respectively. This may result in inconsistencies of perceptions of the behaviors of the same child. Moreover, parents tend to take an ipsative approach when making their evaluations, whereas teachers do normative assessments, i.e., teachers evaluate a child compared to other children. Additional analyses revealed marginal and a non-significant difference between reports of parents and teachers, when both constructs were reported by the same respondent. Hence, the results suggest highly reliable reports of the same respondent, in line with previous studies ([2, 43, 48, 65]. However, still we cannot know whether the same teacher or the same parent reported on both constructs, i.e., whether assessments are highly context dependent rather than person dependent. Furthermore, considering the possible common method variance, our results should still be interpreted with caution.

### Demographic moderators

Percentage of girls in each study was investigated as a continuous moderator on the social competence-behavioral problems relation. Meta-regression analysis did not reveal significant differences for percentage of girls between studies. However, several findings suggest that girls tend to display lower levels of behavioral problems and somewhat higher levels of social competence, compared to boys [10, 61]. Hence, results reflect no gender difference in the strength of the correlation, since both genders will display different levels of social competence and behavioral problems (i.e., girls with high levels of social competence and low levels of behavioral problems versus boys with low levels of social competence and high levels of behavioral problems).

We also examined age as a dichotomous variable, separated as under or over school age (age < 6 vs. age ≥ 6). Age appeared as a significant moderator of the social competence - behavioral problems relation, with a significantly higher correlation among the oldest children. This finding may have several explanations. First, it may reflect that social competence is more easily evaluated and observed among older children, as developmental factors are critical to the assessment of social competence. Furthermore, it may also reflect that measurement instruments are more suitable to capture social competence in older children. Few instruments are developed for children in preschool years, as it is not typically until preschool years that children start engaging in sustained play with other children. Moreover, lack of social competence and behavioral problems are more visible, and have larger consequences, as the child reach school age, which allows children to navigate through different activities and interaction with peer with a fair amount of autonomy [6]. In addition, during school day extracurricular activities are more structured than in preschool years, involving larger peer groups and more coordinated play, in which poor social competences and behavioral problems, become a larger and more visible challenge.

Finally, we investigated whether the mean effect size varied based on study origin. A total of twenty-seven studies were from USA, whereas sixteen studies were European and seven studies were Asian (studies from Canada, Africa and Australia were excluded). Results showed that study origin could not explain variation in the magnitude of the correlation, which imply that the relation between social competence and behavioral problems can be generalized across these continents.

### Limitations, strengths and future studies

The current meta-analysis summarized the various studies on the relation between social competence and behavioral problems among children and adolescents, and tried to answer why the results are heterogeneous in the literature. By using the bandwidth of constructs, we tried to demonstrate whether the strength of this relation varies across constructs or measures used. The results of this meta-analysis provide additional support for targeting multiple areas, rather than just focusing on behavioral problems in targeted interventions.

Several limitations of this study should be considered. First, although we made an effort to identify all eligible studies using different search methods, it is still possible that some studies were not identified by our search strategy. Second, given our defined child age span, we excluded studies that used self-report measures. Self-report studies would also be more relevant when studying older children. The study did not include studies where social competence was rated by parent and behavioral problems by teachers, since these studies comprised less than five studies, which made it not feasible to study their effect as a moderator. Third, the correlations between social competence and behavioral problems may have been influenced by a third variable, whereof internalizing problems may represent a strong candidate [28]. Moreover, the methodological quality of the studies was not considered beyond the fact that all were rated by the authors to have a satifactory quality for inclusion in the study.

## Conclusions

The present meta-analysis examined the strength of the correlation between social competence and behavioral problems in children. Results showed a robust negative correlation of medium effect size between the two constructs. Moderator analyses provided some insight that could explain the large correlational variation among studies (i.e., heterogeneity). The studies where respondents were the same (either parents or teachers) for both social competence and behavioral problems showed significant and high correlations, as compared to those that relied on different respondents. Furthermore, children aged above six showed significantly higher correlations between constructs compared to assessments based on children below age six. Differences related to bandwidth of constructs, instrument type, gender, study origin or socioeconomic status did not seem to change the strength of the social competence – behavioral problems correlation. In conclusion, our results of a robust moderate and negative relation between social competence and these problems should be taken into account when treating children with emerging or present behavioral problems.

## Supplementary information

**Additional file 1.** Search Strategies

## Data Availability

Data are available from the first author Silje Hukkelberg, s.s.hukkelberg@nubu.no

## Abbreviations

ADHD: Attention Deficit Hyperactivity Disorder
APA: American Psychiatric Association
DSM-V: Structured Clinical Interview for DSM Disorders-Fifth Edition
ERIC: Educational Resources Information Center
HCSBS: Home and Community Social Behavior Scales
ICD-10: International Classification of Diseases (ICD) 10th revision
Medline: National Library of Medicine
PRISMA: Preferred Reporting Items for Systematic reviews and Meta-Analyses
SSIS: Social Skills Improvement System
