# Supplementary material for: The relation between behavioral problems and social competence: A correlational Meta-analysis
Source: BMC Psychiatry. 2019 Nov 9;19:354. doi: 10.1186/s12888-019-2343-9 (PMC6842530; doi:10.1186/s12888-019-2343-9)
Supplement: Supplementary file 1 — Additional file 1. Search Strategies [file 12888_2019_2343_MOESM1_ESM.docx]

**Additional file**

**Search Strategies**

Ovid MEDLINE(R) Epub Ahead of Print, In-Process & Other Non-Indexed Citations, Ovid MEDLINE(R) Daily and Ovid MEDLINE(R) 1946 to Present

1 social skills/

2 ((social or interpersonal or inter-personal) adj1 (competenc* or skill* or function*)).tw.

3 1 or 2

4 Problem Behavior/

5 Conduct Disorder/

6 Child Behavior Disorders/

7 Aggression/

8 ((behavio* or conduct or opposition* or defiant* or aggressi*) adj1 (problem* or disorder*)).tw.

9 (externali?ing or misconduct or misbehavio*).tw.

10 ((antisocial or anti-social*) adj1 behavio*).tw.

11 or/4-10

12 3 and 11

13 (child* or young or youth* or adolesc*).tw.

14 12 and 13

15 limit 14 to yr="2008 -Current"

16 (study* or trial*).tw.

17 15 and 16

18 exp study characteristics/

19 15 and 18

20 17 or 19

21 limit 20 to (danish or english or norwegian or swedish or turkish)

Ovid PsycINFO 2002 to June Week 1 2018

1 social skills/

2 ((social or interpersonal or inter-personal) adj1 (competenc* or skill* or function*)).tw.

3 1 or 2

4 exp behavior problems/

5 conduct disorder/

6 Aggressive Behavior/

7 externalization/

8 oppositional defiant disorder/

9 ((behavio* or conduct or opposition* or defiant* or aggressi*) adj1 (problem* or disorder*)).tw.

10 (externali?ing or misconduct or misbehavio*).tw.

11 or/4-10

12 3 and 11

13 (child* or young or youth* or adolesc*).tw.

14 12 and 13

15 limit 14 to yr="2008 -Current"

16 limit 15 to ("0100 journal" or "0110 peer-reviewed journal" or "0400 dissertation abstract")

17 (study* or trial*).tw.

18 15 and 17

19 limit 15 to ("0300 clinical trial" or "0400 empirical study" or "0430 followup study" or "0450 longitudinal study" or "0451 prospective study" or "0453 retrospective study" or 1800 quantitative study)

20 18 or 19

21 limit 20 to ("0100 journal" or "0110 peer-reviewed journal")

22 limit 21 to (danish or english or norwegian or swedish or turkish)

ERIC 1965 to April 2018

1 interpersonal competence/

2 ((social or interpersonal or inter-personal) adj1 (competenc* or skill* or function*)).tw.

3 or/1-2

4 behavior problems/

5 behavior disorders/

6 ((behavio* or conduct or opposition* or defiant* or aggressi*) adj1 (problem* or disorder*)).tw.

7 (externali?ing or misconduct or misbehavio*).tw.

8 or/4-7

9 3 and 8

10 (child* or young or youth* or adolesc*).tw.

11 9 and 10

12 limit 11 to yr="2008 -Current"

13 limit 12 to journal articles

14 limit 13 to (danish or english or norwegian or swedish or turkish)

15 (study* or trial*).tw.

16 14 and 15

17 exp methods/

18 14 and 17

19 16 or 18
